# Chemical characteristics, source apportionment, and regional transport of marine fine particles toward offshore islands near the coastline of northwestern Taiwan Strait

**DOI:** 10.1007/s11356-018-3093-9

**Published:** 2018-09-18

**Authors:** Cheng-Chih Chang, Chung-shin Yuan, Tsung-Chang Li, Yen-Lung Su, Chuan Tong, Shui-Ping Wu

**Affiliations:** 1 Institute of Environmental Engineering, National Sun Yet-sen University, Taiwan, Republic of China; 2 School of Geographic Science, Fujian Normal University, Fujian, China; 3 Center for Marine Environmental Chemistry and Toxicology, College of Environment and Ecology, Xiamen University, Xiamen, China; 4 State Key Laboratory of Marine Environmental Science, Xiamen University, Xiamen, China

**Keywords:** Offshore islands, Marine fine particles (PM_2.5_), Spatiotemporal variation, Chemical characteristics, Transportation routes, Regional transport

## Abstract

This study aims to investigate the spatiotemporal variation, chemical composition, and source apportionment of marine fine particles (PM_2.5_) as well as their regional transport toward the Matsu Islands located near the coastline of northwestern Taiwan Strait. Four offshore island sites located at the Matsu Islands were selected to conduct both regular and intensive sampling of marine PM_2.5_. Water-soluble ionic species, metallic elements, and carbonaceous contents were then analyzed to characterize the chemical characteristics of marine PM_2.5_. In order to identify the potential sources and their contributions to marine PM_2.5_, chemical mass balance (CMB) receptor model was employed along with the backward trajectory simulation to resolve the source apportionment of marine PM_2.5_ and to explore their transport routes in different seasons. The results showed that high PM_2.5_ concentrations were commonly observed during the northeastern monsoon periods. Additionally, marine PM_2.5_ concentration decreased from the west to the east with the highest PM_2.5_ at the Nankang Island and the lowest PM_2.5_ at the Donyin Island in all seasons, indicating an obvious concentration gradient of PM_2.5_ transported from the continental areas to the offshore islands. In terms of chemical characteristics of PM_2.5_, the most abundant water-soluble ions of PM_2.5_ were secondary inorganic aerosols (SO_4_^2−^, NO_3_^−^, and NH_4_^+^) which accounted for 55–81% of water-soluble ions and 29–52% of marine PM_2.5_. The neutralization ratios of PM_2.5_ were always less than unity, indicating that NH_4_^+^ cannot solely neutralize nss-SO_4_^2+^ and NO_3_^−^ in marine PM_2.5_ at the Matsu Islands. Although crustal elements (Al, Ca, Fe, K, and Mg) dominated the metallic content of marine PM_2.5_, trace anthropogenic metals (Cd, As, Ni, and Cr) increased significantly during the northeastern monsoon periods, particularly in winter. Organic carbons (OCs) were always higher than elemental carbons (ECs), and the mass ratios of OC and EC were generally higher than 2.2 in all seasons, implying that PM_2.5_ was likely to be aged particles. During the poor air quality periods, major air mass transport routes were the northern transport and the anti-cyclonic circulation routes. Source apportionment results indicated that fugitive soil dusts and secondary aerosols were the major sources of marine PM_2.5_ at the Matsu Islands, while, in winter, biomass burning contributed up to 15% of marine PM_2.5_. This study revealed that cross-boundary transport accounted for 66~84% of PM_2.5_ at the Matsu Islands, suggesting that marine PM_2.5_ at the Matsu Islands has been highly influenced by anthropogenic emissions from neighboring Fuzhou City as well as long-range transport from Northeast Asia.

## Introduction

The effects of fine particles (PM_2.5_) on human health and ambient air quality have attracted increasing global attentions for the past decades (Li et al. 2016). Primary and secondary PM_2.5_ could be emitted directly from natural and anthropogenic sources as well as chemically formed in the atmosphere, which potentially contains complicated inorganic and organic constituents (Kaneyasu et al. 1995; Kocak et al. 2007; Li et al. 2013a). Exposure to high PM_2.5_ level poses a considerable risk to health problems, such as aggravation of asthma, respiratory symptoms, and even cardiovascular and respiratory diseases (Anderson et al. 1992; Dockery et al. 1993). Long-term exposure to PM_2.5_ is highly associated with an increase in the risk of cardiopulmonary mortality by 6–13% per 10 μg/m^3^ of PM_2.5_ (Beelen et al. 2008; Pope III et al. 2002; Stanek et al. 2011). In addition to human health, high PM_2.5_ concentration could cause poor ambient air quality, atmospheric visibility attenuation (i.e., regional haze), and global climate change and interfere with the earth’s radiation balance due to its complicated physicochemical characteristics of atmospheric PM_2.5_. Chemical composition (e.g., SO_4_^2−^ and NO_3_^−^) and size distribution (0.4–0.9 μm) of PM_2.5_ play a crucial role in atmospheric visibility degradation (Yuan et al. 2002; Lee et al. 2005; Yuan et al. 2006).

Recently, more and more countries have regulated PM_2.5_ as a criteria air pollutant in the ambient air quality standards, whereas PM_2.5_ has been monitored in a regular basis for local, regional, and global scales. In particular, intensive sampling of atmospheric PM_2.5_ has been widely undertaken in the highly polluted metropolitan and industrial areas. However, they are mostly undertaken in the lands and seldom conducted in the seas or at the islands. For the past decades, China has experienced rapid economic and industrial development and thus significantly increases its fossil fuel consumption and anthropogenic emissions of particles, resulting in severe environmental problems, particularly poor ambient air quality and human health. The Matsu Islands situated at the Minjiang Estuary faces Fuzhou City in the southeastern coast of China. The Matsu Islands have absolutely no industrial and mobile sources, which conserved it as a background environment in a long-term period. However, its ambient air quality became poor recently and in some occasions even worse than the ambient air quality of major cities in Taiwan.

The Matsu Islands are located in the northern margin of the subtropical monsoon weather with an annual average temperature of about 19.3 °C and uneven seasonal variation of rainfall. The prevailing winds are blown from the northeast from October to May of sequential year, while the southwestern monsoons occur mainly from June to September. The Matsu Islands have only a population of about 11,000 and a total area of 29.5 km^2^ and has no large-scale anthropogenic sources. Major stationary sources at the Matsu Islands include a 14.2-MW diesel-fired utility power plant, two breweries, and a few gas stations. However, high PM_10_ concentration up to 129 μg/m^3^ has been recorded by Taiwan EPA’s ambient air quality monitoring station located at the Nankang Island in the spring of 2011 (Liao et al. 2018).

Previous atmospheric PM_10_ concentration measured at the Matsu Islands showed that PM_10_ concentration in summer was always the lowest in the whole year, while high PM_10_ concentrations were generally observed during the periods from late fall to early spring of the sequential year (Liao et al. 2018). The temporal variation of PM_10_ concentration correlated with wind direction in the marine boundary layer which was closely related with poor ambient air quality (Pollutants Standard Index, PSI > 100) during the prevalence of northeastern monsoons commonly occurs in the seasons of winter and spring. However, there are very few PM_2.5_ sampling campaigns that have been conducted at offshore islands such as the Matsu Islands all over the world. The chemical characteristics and source apportionment of marine PM_2.5_ as well as the influences of regional transport on marine PM_2.5_ have not been thoroughly investigated so far.

Accordingly, this study aims to investigate the spatiotemporal variation and chemical characteristics of marine PM_2.5_ at the Matsu Islands. The source identification and apportionment of marine PM_2.5_ were further determined by a CMB receptor model and backward trajectory simulation. The percentages of cross-boundary transport (CBT) contributed to marine PM_2.5_ at the Matsu Islands in different seasons were further estimated.

## Experimental methods

### Sampling methods and site description

**Fig. 1:**
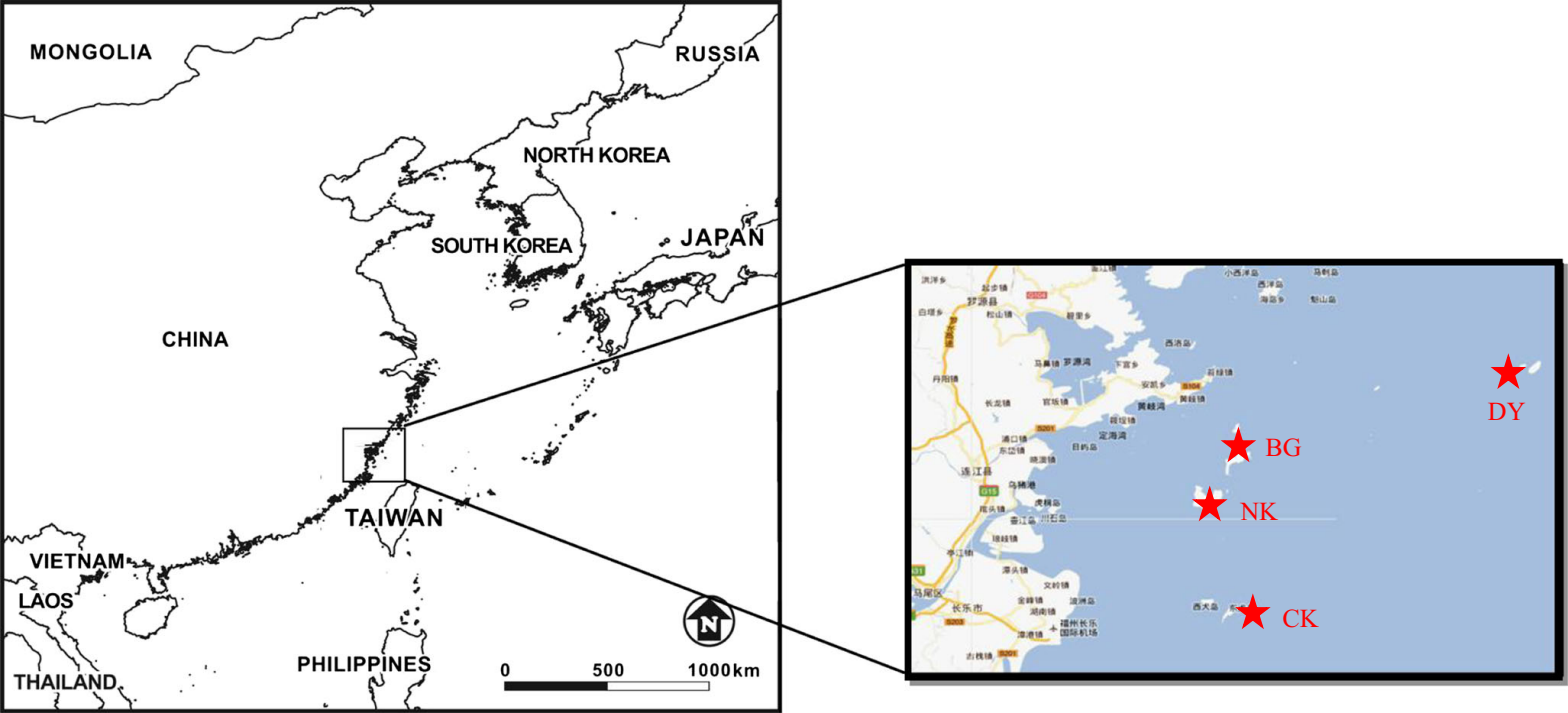
Location of PM_2.5_ sampling sites at the Matsu Islands in the northeastern Taiwan Strait

Marine PM_2.5_ was simultaneously sampled at four offshore island sites at the Matsu Islands including Nankang (NK) (119°55′N, 26°10′E), Beigang (BG) (119°58′N, 26°13′E), Donyin (DY) (120°29′N, 26°21′E), and Chiukung (CK) (119°93′N, 25°97′E) townships (see Table 1 and Fig. 1). In this study, a PM_2.5_ sampler (BGI, PQ-200) was applied to collect marine PM_2.5_ at each sampling site. Both regular and intensive samplings of marine PM_2.5_ were conducted from July 2013 to May 2014. Regular sampling was conducted to simultaneously collect 24-h PM_2.5_ (from 8:00 am to 8:00 am of sequential day) at four sampling sites once every 8 days excluding the rainy days. It means that PM_2.5_ samples would not be collected at all four sampling sites if it rained at any sampling site due to the limitation of ferry transportation to the four island sites as well as the shortage of manpower to rearrange the complementary sampling of PM_2.5_ in the following few days. Intensive sampling was undertaken to simultaneously collect 24-h PM_2.5_ at four island sites for five consecutive days on November 18–22, 2013. Intensive sampling mainly focused on increasing the temporal frequency of PM_2.5_ sampling during a typical PM_2.5_ episode occurred at the Matsu Islands. As a result, a total of 39 PM_2.5_ samples were collected at each island site in the Matsu Islands, including 34 regular samples and 5 intensive samples (see Table 3).

**Table 1:** Location and environmental description of PM_2.5_ sampling sites at the Matsu Islands

Sites	Sampling location	Sample no. (*n*)	Latitude	Longitude	Altitude (m)	Site description
NK	Nankang	34	26°10′09E	119°55′25N	42	Hillside at Nankang Island
BG	Beigang	34	26°13′27E	119°58′48N	38	Hillside at Beigang Island
DY	Donyin	34	26°21′51E	120°29′44N	31	Open area at Donyin Island
CK	Chiukung	34	25°97′47E	119°93′64N	35	Hillside at Chiukung Island

The regular sampling was conducted once every 8 days exclusive rainy days, meaning no PM_2.5_ samples were taken at all four sampling sites if it rained at any sampling site

The concentration contours of PM_2.5_ over the Matsu Islands in four seasons was plotted respectively based on the seasonal average concentrations of PM_2.5_ sampled at four island sites (see Table 3) by a spatial plotting software (SURFER) which has been widely used to describe the spatial distribution of air pollutants for metropolitan district, industrial areas, and rural areas (Otvos et al. 2003; Shaocai et al. 2004; Tsai et al. 2010).

### Analytical methods of chemical composition

Posterior to field sampling of marine PM_2.5_, quartz fiber filters were temporarily stored at 4 °C and then transported back to the Air Pollution Laboratory in the Institute of Environmental Engineering at National Sun Yat-sen University for further conditioning, weighing, and chemical analysis. Each quartz fiber filter was cut into four identical portions for further analyzing the chemical composition of PM_2.5_. Among them, one quarter of the filter was analyzed for water-soluble ionic species by putting it inside a 15-mL polypropylene (PE) bottle and pouring the distilled de-ionized water (D.I. H_2_O) into each bottle for dissolving ions in an ultrasonic vibration process for at least 60 min. An ion chromatography (Dionex, Model DX-120) was used to analyze the concentration of major anions (F^−^, Cl^−^, SO_4_^2−^, and NO_3_^−^) and cations (NH_4_^+^, K^+^, Na^+^, Ca^2+^, and Mg^2+^) of marine PM_2.5_.

Another quarter of the quartz fiber filter was initially digested in a 20-mL mixed acid solution (HNO_3_/HClO_3_ = 3:7) at 150–200 °C for at least 2 h, and then diluted to 25 ml with distilled de-ionized water (D.I. H_2_O) for metallic element analysis. The metallic elements of PM_2.5_ including Cr, Mn, Fe, Ni, Zn, Cd, Pb, Mg, K, Ca, Ti, Al, and As were then analyzed with an inductively coupled plasma-atomic emission spectrometer (ICP-AES) (Perkin Elmer, Model Optima 2000DV).

By cutting one quarter of the filter into two identical parts, two eights of the filters were further used to measure the carbonaceous content of marine PM_2.5_. The carbonaceous content including elemental, organic, and total carbons (OC, EC, and TC) were measured with an elemental analyzer (EA) (Carlo Erba, Model 1108). Before weighing, all quartz fiber filters were baked at 900 °C for 1.5 h to remove carbon impurities from the filters. The preheating procedure would minimize the background carbon in the quartz fiber filter matrix, which might cause interference with the analytical results, leading to a possible overestimation of the carbonaceous content of PM_2.5_. The elemental analyzer was operated in the procedure of oxidation and reduction at 1020 and 500 °C, respectively, with continuous heating for 15 min. Among these two filters, one eighth of the filter was heated in advance using nitrogen gas at 340–345 °C for at least 30 min to expel the organic carbon (OC) fraction from the filters, after which the amount of elemental carbon (EC) was determined. Another eighth of the quartz fiber filter was analyzed without heating to determine the total carbon (TC) of PM_2.5_. As a result, the amount of OC was then estimated by subtracting EC from TC.

### Quality assurance and quality control

The quality assurance and quality control (QA/QC) for both PM_2.5_ sampling and chemical analysis were further conducted in this study. Prior to conducting PM_2.5_ sampling, the flow rate of each PM_2.5_ sampler was calibrated with a film flowmeter (Sensidyne, Model MCH-01). Quartz fiber filters were then carefully handled and placed on the PM_2.5_ samplers to prevent potential interferences during the sampling procedure. After sampling, aluminum foil was used to fold the sampled quartz fiber filters, which were then temporarily stored at 4 °C and transported back to the Air Pollution Laboratory in the Institute of Environmental Engineering at National Sun Yat-sen University for further chemical analysis.

The sampling and analytical procedure was similar to that described in various previous studies (Cheng and Tsai 2000; Lin, 2002; Yuan et al. 2006; Tsai et al. 2010). Both field and transportation blanks were undertaken for PM_2.5_ sampling, while reagent and filter blanks were applied for chemical analysis. The field blanks were exposed in the field when the quartz fiber filters were placed and removed from the PQ-200 samplers. Background contamination was routinely monitored by using operational blanks (unexposed filters), which were proceeded simultaneously with field samples. In this study, the field blanks were extremely low, normally below or close to the method detection limit. Therefore, the background contamination/interference was insignificant and can thus be ignored. The determination coefficient (*R*^2^) of the calibration curve for each chemical species analyzed is required to be higher than 0.995. At least 10% of the samples were analyzed by spiking with a known amount of metallic and ionic species to determine their recovery percentages.

### Chemical transformation of SO_2_ and NO_X_

Secondary inorganic compounds including sulfate and nitrate are the major components contained in the fine particles. To determine the degree of atmospheric transformation of SO_2_ to SO_4_^2−^ and NO_x_ to NO_3_^−^, respectively, the sulfur and nitrogen oxidation ratios (i.e., SOR and NOR) were employed, and these ratios are defined as follows (Colbeck and Harrison 1984; Ohta and Okita 1990):

1 $$ \mathrm{SOR}=\frac{S_{\mathrm{nss}-{{\mathrm{SO}}_4}^{2-}}}{S_{\mathrm{nss}-{{\mathrm{SO}}_4}^{2-}}+{S}_{{\mathrm{SO}}_2}} $$

2 $$ \mathrm{NOR}=\frac{N_{{{\mathrm{NO}}_3}^{-}}}{N_{{{\mathrm{NO}}_3}^{-}}+{N}_{{\mathrm{NO}}_2}} $$

where nss-SO_4_^2−^ is the excess non-sea salt sulfate that was calculated by subtracting the amount of SO_4_^2−^ of marine particles from that of SO_4_^2−^ in the particles (Kaneyasu et al. 1995; Cheng et al. 2000). The units of *S*_nss-SO42−_, ${S}_{{\mathrm{SO}}_2}$, ${N}_{{{\mathrm{NO}}_3}^{-}}$, and ${N}_{{\mathrm{NO}}_2}$ are neq/m^3^. The average concentrations of SO_2_ and NO_x_ during each sampling periods were obtained from the ambient air quality monitoring station nearby the sampling site at the Matsu Islands.

### CMB receptor model

The source apportionment of PM_2.5_ could be assessed by using a receptor model based on the principle of chemical mass balance (CMB) (Ke et al. 2007; Kothai et al. 2008; Yatkin and Bayram 2008; Wang et al. 2008). Since the detailed descriptions of CMB receptor model (i.e., CMB8) are available elsewhere, only a brief summary is presented herein.

The CMB receptor model uses the emission profiles of prominent sources to estimate their contributions to a specific receptor. It is assumed that the total concentration of a particular chemical species at the receptor site is the linear summation of each individual contribution from various sources. The CMB receptor model uses the results of the least-square regression analysis of the chemical composition of PM_2.5_ to estimate the most appropriate contributions of emission sources. Therefore, the CMB receptor model resolves a least-square solution to a set of linear equations. The solution expresses each receptor’s concentration of a chemical species as a linear summation of the products of source profiles and source contributions. In this study, the input parameters for CMB receptor modeling included 24 chemical species of PM_2.5_ (see Figs. 5, 9, and 10) and source profiles covering 32 potential sources (see Table 2). Source profiles (the fractional amount of each species in the emissions from each source type) and receptor concentrations, each with realistic uncertainty estimates, serve as input data to the CMB receptor model. The model output consists of the contribution from each source type to the total ambient aerosol mass, as well as to the concentrations of individual chemical species. The CMB receptor modeling results are further evaluated by several fit indices, such as *R*^2^ (≥ 0.8), *χ*^2^ (≤ 4.0), *T* statistics (≥ 2.0), and mass percentages accounting for 80–120%. The source profiles used in this study have been reported by USEPA and Southern California Air Quality Study and research data obtained from the investigation on the chemical composition of PM_2.5_ and the source profiles of primary sources in Taiwan (see Table 2).

**Table 2:** Source profiles of PM_2.5_ used for chemical mass balance receptor modeling (Li et al. 2013a, b)

Source no.	Codes	Sources	Researchers
SCT004	PBPRI1	Petroleum cracking plant	U.S. EPA (1991)
SCT007	PP004	Industrial boilers (Oil)	Cheng et al. (2001)
SCT008	PP005	Industrial boilers (Coal)	Cheng et al. (2001)
SCT009	PETRO1	Petroleum industry	U.S. EPA (1991)
SCT010	STEEL1	Steel industry	Chiang et al. (1993)
SCT011	STEEL2	Coke plants	Chiang et al. (1993)
SCT012	STEEL3	Sinter plants	Chiang et al. (1993)
SCT013	STEEL4	Electric arc furnaces	Yuan et al. (2003)
SCT020	CEMENT	Cement industry	Chiang et al. (1993)
SCT023	VEHICLE2	Vehicular exhausts	Chow, J.C. (1991)
SCT024	VEHICLE3	Diesel exhausts	Chow, J.C. (1991)
SCT025	DUST1	Paved road dust in South Taiwan	Cheng et al. (1998)
SCT026	DUST2	Paved road dust in Central Taiwan	Cheng et al. (1998)
SCT027	DUST3	Paved road dust in South Taiwan	Yuan et al. (1991)
SCT028	DUST4	Paved road dust in Central Taiwan	Chiang et al. (1993)
SCT029	DUST5	Unpaved road dust in Central Taiwan	Chiang et al. (1993)
SCT031	SOIL1	Soil dust	U.S. EPA (1991)
SCT033	MARIN1	Marin in Central Taiwan	Cheng et al. (1998)
SCT034	MARIN2	Marin in South Taiwan	Chen et al. (1998)
SCT035	VB001	Biomass burning	Cheng et al. (1999)
SCT037	SO4	Secondary sulfate	Wang et al. (2006)
SCT038	NO2	Secondary nitrate	Wang et al. (2006)
SCT039	STONE	Stone processing industry	Li et al. (2013b)
SCT040	CEMENT2	Cement industries	Li et al. (2013b)
SCT041	CERM1	Ceramic plants	Li et al. (2013b)
SCT042	CERM2	Tile industries	Li et al. (2013b)
SCT043	COAL	Coal burning	Li et al. (2013b)
SCT044	COAA	Coal ash	Li et al. (2013b)
SCT045	SOIL2	Fugitive dust	Li et al. (2013b)
SCT046	VB002	Biomass burning	Li et al. (2013b)
SCT047	CONST	Construction dust	Li et al. (2013b)
SCT048	DUST6	Road dust	Li et al. (2013b)

The source profiles used in this study were mainly obtained from the researcher’s finding of the chemical composition of PM_10_ emitted from various emission sources. Only limited source profiles are referred from USEPA and Southern California Air Quality Study and local emission source profiles

## Results and discussion

### Spatiotemporal variation of PM_2.5_ at the Matsu Islands

**Fig. 2:**
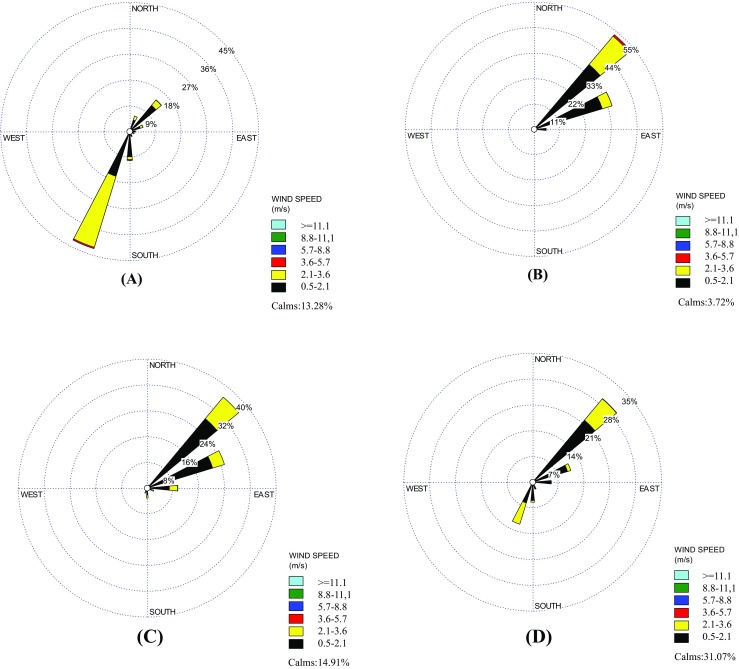
Seasonal variation of wind direction during the sampling periods (**a** summer, **b** fall, **c** winter, **d** spring)

**Fig. 3:**
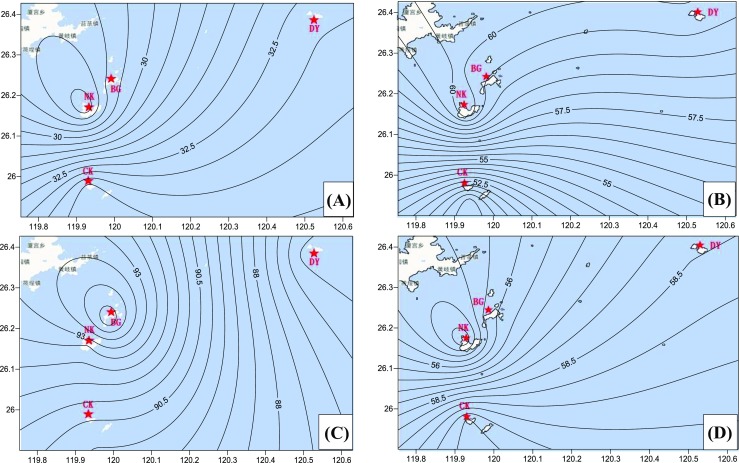
Seasonal variation of PM_2.5_ concentration contours at the Matsu Islands (**a** summer, **b** fall, **c** winter, **d** spring)

The spatial distribution and temporal variation of PM_2.5_ concentration over the target region were determined and plotted by the concentrations of marine PM_2.5_ sampled at four island sites in the Matsu Islands. Figures 2 and 3 illustrate the seasonal variation of wind speed, wind direction, and PM_2.5_ concentration contours. On the whole, field sampling results indicated that low monthly average PM_2.5_ concentrations were commonly observed in summer, while high monthly average PM_2.5_ concentrations mostly occurred in winter and early spring (see Table 3). It was further found that a maximum PM_2.5_ concentration level of 82.6 μg/m^3^ was observed during the northeastern monsoon periods, which was approximately 9.5 times higher than the lowest PM_2.5_ concentration of 8.7 μg/m^3^. From the perspective of spatial distribution, PM_2.5_ concentrations had a tendency to decrease from the west coastline to the easternmost island (site DY) in all seasons. Moreover, PM_2.5_ concentrations decreased from the north to the south in fall and winter, while an opposite trend was observed in spring and summer. In particular, PM_2.5_ concentrations observed at DY were always the lowest while compared to other three sampling sites in the Matsu Islands. It is worth noting that the spatial distribution of marine PM_2.5_ was, however, inconsistent with the seasonal prevailing wind direction, suggesting that marine PM_2.5_ at the Matsu Islands might not be solely influenced by long-range transport, but also by other factors, such as local anthropogenic emissions from neighboring Fuzhou City, which has been pointed out in our previous findings at the Kinmen Islands (Li et al. 2013a, b).

**Table 3:** Seasonal variation and spatial distribution of marine PM_2.5_ concentrations in the Matsu Islands

Sampling sites	Summer (*n* = 28)	Fall (*n* = 52^a^)	Winter (*n* = 40)	Spring (*n* = 36)
Nankang (NK)	17.07 ± 9.87	27.49 ± 12.97	47.91 ± 21.18	41.65 ± 15.14
Beigang (BG)	17.93 ± 8.30	26.79 ± 13.00	46.31 ± 19.88	39.73 ± 14.06
Donyin (DY)	16.57 ± 7.29	22.83 ± 12.53	42.44 ± 20.69	35.17 ± 14.78
Chiukung (CK)	17.01 ± 6.92	24.10 ± 10.48	44.17 ± 18.71	36.40 ± 13.83
Spatial average	17.14 ± 7.64	25.30 ± 12.15	45.21 ± 19.25	38.24 ± 13.74

^a^A total of 32 regular samples and 20 intensive samples were collected in Fall

**Fig. 4:**
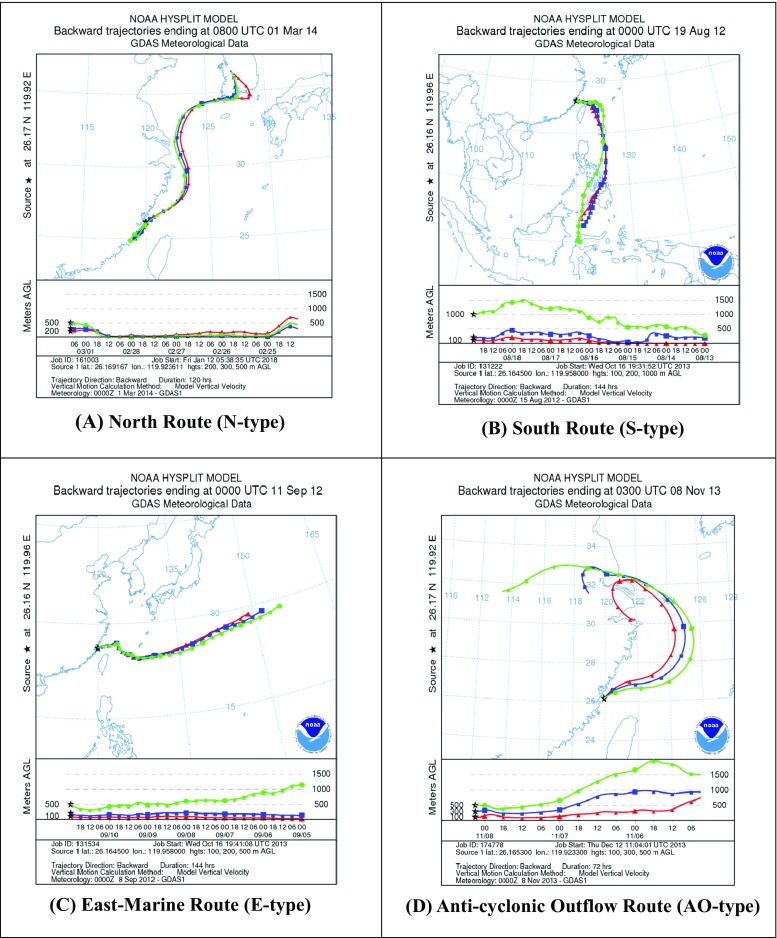
Four typical routes of air parcels transported toward the Matsu Islands

In addition, to plot the contours of PM_2.5_ concentration in a domain of interest, an air trajectory scheme was further applied to track the transport routes of air masses moving toward the Matsu Islands. In general, hybrid single-particle Lagrangian integrated trajectory (HYSPLIT) is a commonly used track model to plot both backward and forward trajectories of an air parcel moved to a specific site at various heights aboveground over a period of time. In this study, the backward trajectories were plotted for 120 h in four typical routes as illustrated in Fig. 4. The routes were drawn starting from a site of 119°44′N, 26°10′E with the heights of 100, 200, and 300 m, respectively, to track the transport routes of air parcels arriving at the Matsu Islands. As far as the transport routes were concerned, PM_2.5_ concentrations of the northern routes mainly during the northeastern monsoon periods were mostly higher than those of the southern routes. Accordingly, the concentrations of marine PM_2.5_ were highly influenced by meteorological conditions, particularly the prevalent winds. We also found that the concentrations of marine PM_2.5_ in spring, fall, and winter were much higher than those in summer.

### Chemical composition of PM_2.5_ at the Matsu Islands

**Fig. 5:**
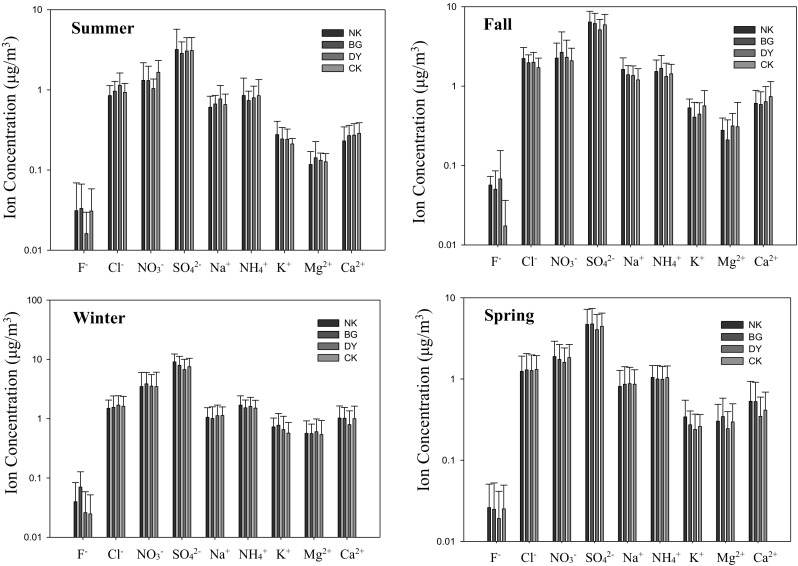
Seasonal variation of water-soluble ionic concentration for marine PM_2.5_ sampled at the Matsu Islands

The seasonal variation of water-soluble ionic species (WSIs) of marine PM_2.5_ sampled at the Matsu Islands is illustrated in Fig. 5. It showed that the most abundant water-soluble ionic species of PM_2.5_ were SO_4_^2−^, NO_3_^−^, and NH_4_^+^, indicating that the probable chemical component of PM_2.5_ was secondary inorganic aerosols (SIAs) composed of ammonium sulfate ((NH_4_)_2_SO_4_) and ammonium nitrate (NH_4_NO_3_), which concurred with previous studies (Yao et al. 2003; Han et al. 2007; Kocak et al. 2007; Tsai et al. 2012). Overall, SIAs accounted for as high as 55–81% of WSIs and 29–52% of marine PM_2.5_, indicating that marine PM_2.5_ at the Matsu Islands were contributed not solely from oceanic sprays but also from secondary aged particles originated from inland anthropogenic sources.

**Fig. 6:**
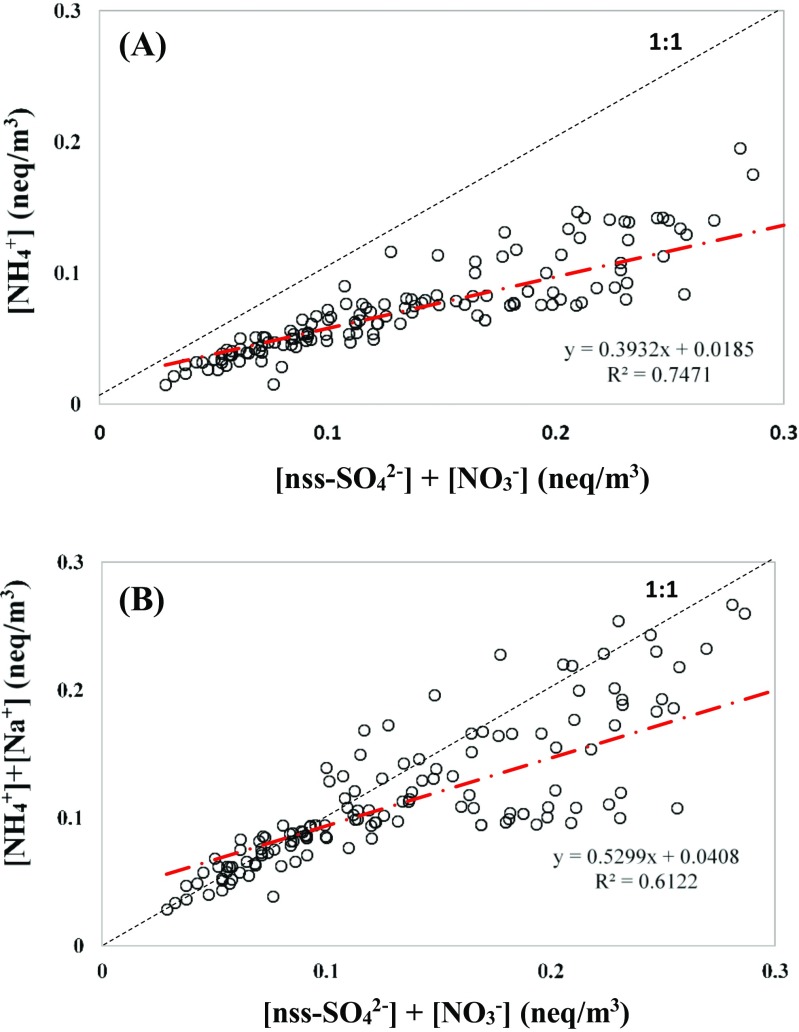
The neutralization ratios of **a** [NH_4_^+^] to [nss-SO_4_^2-]^ + [NO_3_^−^] and **b** [NH_4_^+^] + [Na ^+^] to [nss-SO_4_^2−^] + [NO_3_^−^] for marine PM_2.5_ sampled at the Matsu Islands

**Fig. 7:**
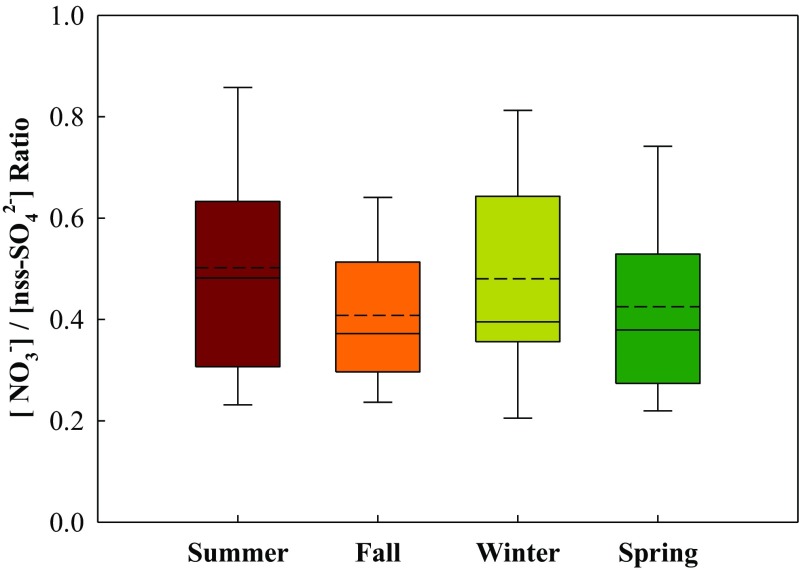
Seasonal variation of [NO_3_^−^]/[nss-SO_4_^2−^] ratio of marine PM_2.5_ sampled at the Matsu Islands

Figure 6 illustrates the neutralization of nss-[SO_4_^2−^] + [NO_3_^−^] by [NH_4_^+^] or [NH_4_^+^] + [Na^+^]. Neutralization ratios (NRs), defined as the equivalent ratio of [NH_4_^+^]/(nss-[SO_4_^2−^] + [NO_3_^−^]) in PM_2.5_, were generally lower than unity, indicating that PM_2.5_ were mainly acidic particles and NH_4_^+^ cannot solely neutralize both nss-SO_4_^2−^ and NO_3_^−^. Furthermore, as shown in Fig. 7, the equivalent ratios of nitrate to non-sea salt sulfate ([NO_3_^−^]/nss-[SO_4_^2−^]) in all seasons ranged from 0.21 to 0.84, suggesting that these acidic fine particles were mainly attributed from sulfur-rich fuel burning sources, i.e., heavy oil burning from industrial boilers and coal burning from coal-fired power plants. As for the NRs of PM_10_ at the Matsu Islands, they were slightly lower than those at the inland sites although their differences were insignificant (Li et al. 2013a). Previous study reported that oceanic sprays seem to be influential to marine PM_2.5_ at the offshore islands while compared to those at the in-land regions (Lee et al. 2005). In this study, Na^+^ is thought to be taken into consideration at the Matsu islands since Na^+^ could play an acidic neutralizer.

**Fig. 8:**
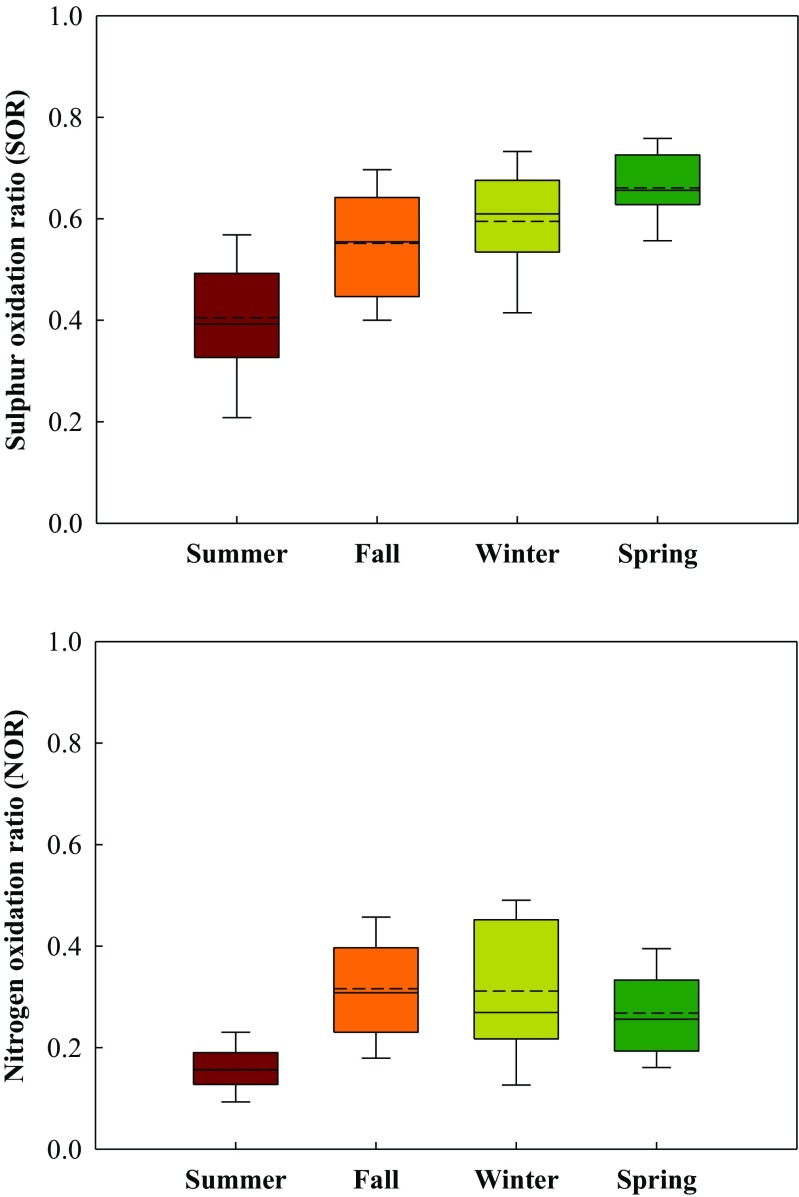
Seasonal variation of SOR and NOR for marine PM_2.5_ sampled at the Matsu Islands

Sulfur and nitrogen oxidation ratios (i.e., SOR and NOR) were further applied to determine the degrees of atmospheric chemical transformation of SO_2_ to SO_4_^2−^ and NO_2_ to NO_3_^−^, respectively. These two ratios can be used to identify the existence and extent of aged secondary inorganic aerosols. Figure 8 illustrates the SOR and NOR of PM_2.5_ sampled at the Matsu Islands. As reported in previous studies, SOR and NOR lower than 0.25 and 0.10, respectively, are thought as primary particles, while SOR and NOR higher than 0.25 and 0.10, respectively, are secondary particles (Ohta and Okita 1990). This study found that the SOR and NOR of marine PM_2.5_ were mostly higher than 0.25 and 0.1, respectively, indicating that marine PM_2.5_ was mainly aged secondary inorganic aerosols (SIAs). These results showed that PM_2.5_ sampled at the Matsu Islands were highly influenced by cross-boundary transport rather than local emissions. Furthermore, the SOR of PM_2.5_ was higher than the NOR of PM_2.5_, indicating that secondary sulfate containing particles formed by SO_2_ contributed more than secondary nitrate containing particles formed by NO_2_ in marine PM_2.5_ sampled at the Matsu Islands.

**Fig. 9:**
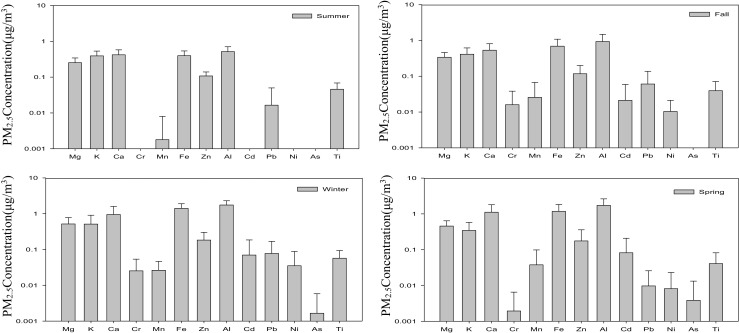
Seasonal variation of metallic elemental concentration for marine PM_2.5_ sampled at the Matsu Islands

To further identify whether the metallic elements in marine PM_2.5_ at the Matsu islands were primarily from natural or anthropogenic processes, the enrichment factor (EF) of each metallic element was determined and applied as the first step in evaluating the influences of crustal sources on the components of atmospheric PM_2.5_ (Sailesh and Mukesh 2010; Chester et al. 2000). The seasonal variation of metallic elements in PM_2.5_ sampled at four island sites is illustrated in Fig. 9. It showed that crustal metals (i.e., Al, Fe, Ca, and Mg) contributed the major portion of metallic content in PM_2.5_ in all seasons, while trace anthropogenic metals (i.e., Cd, As, Ni, and Cr) appeared mainly during the northeastern monsoon periods (i.e., fall, winter, and spring), showing that the northeastern monsoons could blow fine particles (PM_2.5_) containing trace metals from the upwind source regions to the Matsu Islands. Al, Fe, and Ca are the main metallic elements in the earth’s crustal particles which are probably emitted from wind-blown dust, paved and unpaved roads, cement plants, and so on. However, anthropogenic metals could be transported toward the Matsu Islands through cross-boundary transport during the periods of northeastern monsoons.

**Fig. 10:**
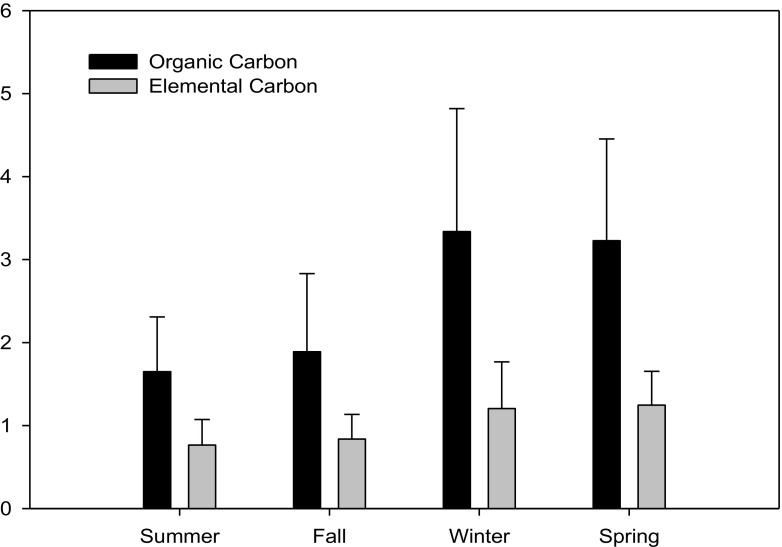
Seasonal variation of carbonaceous concentration for marine PM_2.5_ sampled at the Matsu Islands

Finally, Fig. 10 illustrates the seasonal variation of carbonaceous contents including organic, elemental, and total carbons (i.e., OC, EC, and TC) of marine PM_2.5_ sampled at the Matsu Islands. It showed that the seasonal average concentrations of total carbons were ordered as winter > spring > fall > summer. Overall, it was observed that the mass concentrations of TC in PM_2.5_ in winter and spring were higher than those in fall and summer. Additionally, the concentrations of OC in PM_2.5_ were always higher than those of EC in four seasons at all sampling sites. These findings were in accordance with previous literature, reporting that elemental carbon (EC) originates primarily from direct emissions from combustion sources, while organic carbon (OC) is mainly emitted from either primary anthropogenic sources or secondary organic aerosols that are chemically formed in the atmosphere (Gray et al. 1986; Turpin et al. 1990). Furthermore, the seasonal average mass ratios of OC to EC (OC/EC) ranged from 2.2 to 2.8 which were mostly higher than 2.2 (Gray et al. 1986; Hildemann et al. 1991), showing that PM_2.5_ sampled at the Matsu Islands was also highly influenced by aged secondary organic aerosols (SOAs) during the sampling periods. As far as the seasonal variation, the mass ratios of OC/EC in winter and spring were commonly higher than those in fall and summer.

### Source apportionment of PM_2.5_ at the Matsu Islands

**Fig. 11:**
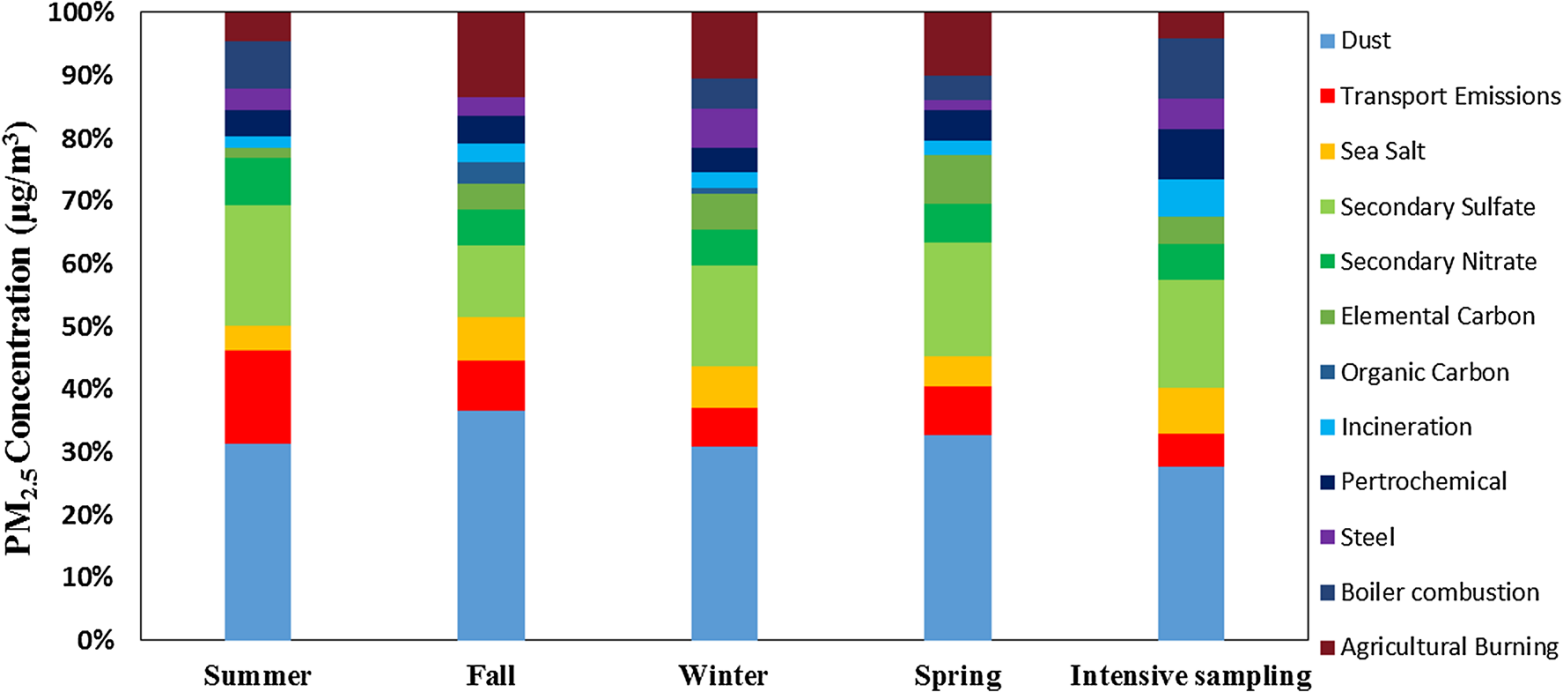
Contribution percentages of potential sources for regular and intensive sampling periods of marine PM_2.5_ sampled at the Matsu Islands

The types of potential sources and their contributions to marine PM_2.5_ at the Matsu Islands was further resolved by using a receptor model based on chemical mass balance (i.e., CMB8.0) (Ke et al. 2007; Kothai et al. 2008; Wang et al. 2008; Yatkin and Bayram 2008). Figure 11 and Table 4 show the source apportionment of PM_2.5_ during the regular and intensive sampling periods at the Matsu Islands. Although the source types and their contribution percentages varied with seasons, the major sources of PM_2.5_ at the Matsu Islands were soil dusts, secondary sulfate and nitrate aerosols, and agricultural debris burning (i.e., biomass burning). Oceanic spray (i.e., sea salts) and vehicular exhausts contributed about 8% of PM_2.5_, respectively. Additionally, the contribution of agricultural debris burning (i.e., biomass burning) could rise up significantly to as high as 9–13% of PM_2.5_ in fall, winter, and spring, reflecting that the open burning of agricultural debris was frequently occurred at the Matsu Islands in these three seasons. It is also worth noting that, during the northeastern monsoon periods, air parcels were mainly transported northerly toward the Matsu Islands due to the dominant high-pressure anti-cyclonic circulation system centering in the Mongolian Plateau. Air parcels blown from northern and northeastern China could migrate downwards along the eastern coastline of China, passing through major metropolitan areas and industrial complexes in the Yangtze River Delta (YRD) and several rapidly developing regions such as Shandong, Jiangsu, and Zhejiang Provinces in the coastal regions of southeastern China.

**Table 4:** The contribution of potential sources for regular and intensive sampling of marine PM_2.5_ at the Matsu Islands

Sources	Regular sampling				Intensive sampling
	Summer	Fall	Winter	Spring	
Incineration plants	1.51%	2.79%	2.15%	2.22%	7.08%
Petroleum plants	3.40%	4.22%	3.15%	4.66%	5.32%
Steel plants	2.82%	2.86%	5.30%	1.69%	1.64%
Industrial boilers (Oil)	6.31%	–	3.94%	1.41%	4.15%
Coal-fired boiler	–	–	–	2.30%	–
Vehicular exhausts	12.44%	7.71%	5.09%	7.75%	3.56%
Street dust	26.05%	35.50%	25.95%	32.07%	31.50%
Oceanic spray	3.17%	6.67%	5.73%	4.66%	8.34%
Secondary sulfate	16.02%	10.99%	13.35%	17.73%	19.87%
Secondary nitrate	6.27%	5.66%	4.84%	6.12%	6.58%
Secondary organic carbon	1.37%	3.95%	4.82%	7.59%	4.88%
Secondary elemental carbon	–	3.34%	0.83%	–	–
Biomass burning	3.76%	13.13%	8.91%	9.99%	5.01%

– no contribution to marine PM_2.5_

During the regular sampling periods, secondary sulfate and nitrate, fugitive dusts, biomass burning, vehicular exhausts, sea salts, petrochemical plants, iron works, and waste incinerators contributed 20.2%, 29.9%, 8.9%, 8.2%, 5.1%, 3.9%, 3.2%, and 2.2% of marine PM_2.5_, respectively. Moreover, during the intensive sampling periods, secondary sulfate and nitrate, fugitive dusts, sea salts, waste incinerators, petrochemical plants, biomass burning, organic carbon, and vehicular exhausts contributed 26.4%, 31.5%, 8.3%, 7.1%, 5.3%, 5.0%, 4.9%, and 3.56% of PM_2.5_, respectively. It shows that the contribution percentages of secondary sulfate and nitrate, fugitive dusts, sea salts, waste incinerators, and petrochemical plants during the intensive sampling periods were higher than those during the regular sampling periods.

### CBT of PM_2.5_ at the Matsu Islands

This study further estimated the contribution percentages of cross-boundary transport (CBT) for marine PM_2.5_ at the Matsu Islands. The cross-boundary transport of PM_2.5_ was defined as the PM_2.5_ emitted from the upwind sources and migrated toward the Matsu Islands. During the sampling periods of this particular study, the lowest PM_2.5_ concentration of 8.70 μg/m^3^ measured in an annual basis can be treated as the background level of PM_2.5_ at the Matsu Islands by assuming that the variation of mixing layer height and meteorological conditions were somewhat negligible. The deduction of the background PM_2.5_ concentration from the field measured PM_2.5_ concentrations can be assumed as the contributions of CBT to PM_2.5_ transported toward the Matsu Islands.

**Fig. 12:**
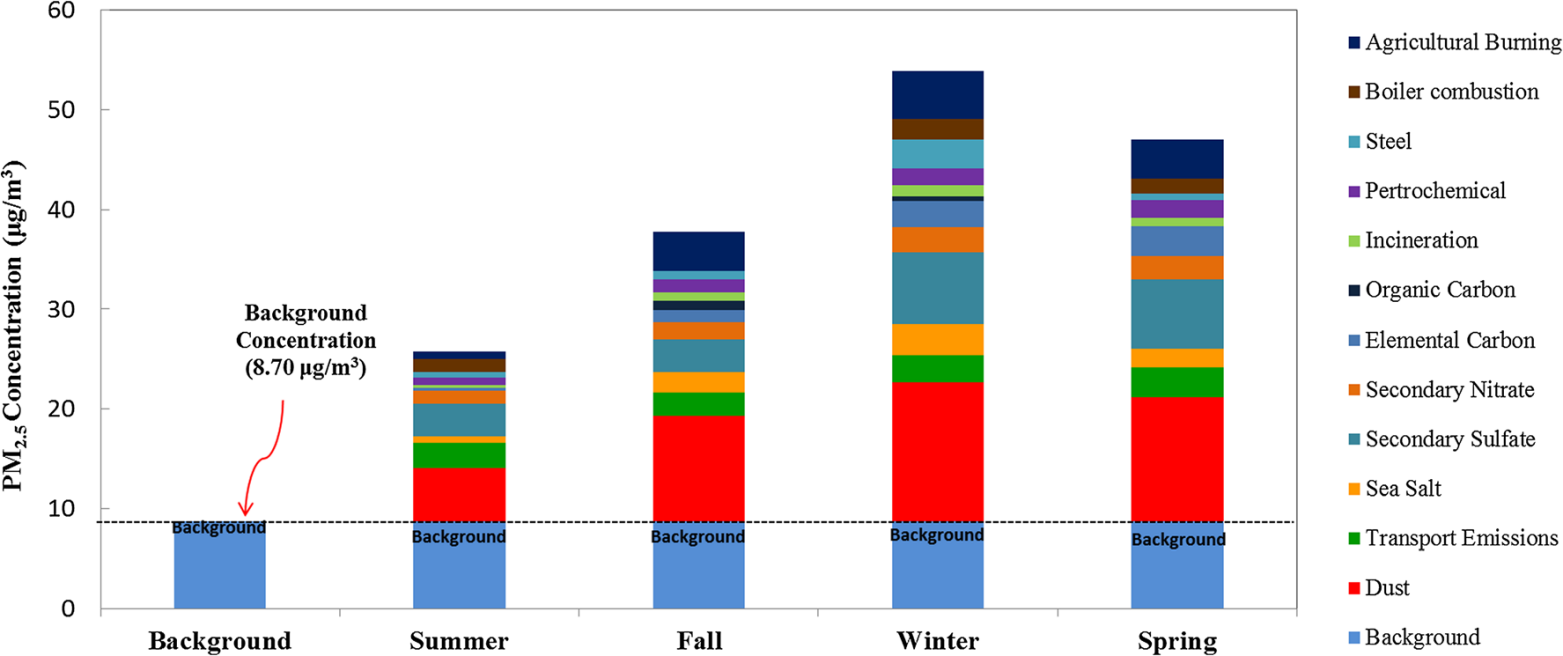
Seasonal variation of cross-boundary contribution to marine PM_2.5_ sampled at the Matsu Islands

Figure 12 illustrates the contribution percentages of various sources of PM_2.5_ transported toward the Matsu Islands in different seasons. It showed that secondary sulfate and nitrate aerosols were mainly attributed from the dramatic increase of cross-boundary transport. Similar trends were also observed for industrial processes, soil dusts, and carbonaceous materials. The overall contribution percentages of cross-boundary transport to PM_2.5_ at the Matsu Islands in summer, fall, winter, and spring were estimated as 66.3%, 77.0%, 83.9%, and 81.5%, respectively, which were higher than those at the Penghu Islands (36.4–76.8%), the Dongsha Islands (26.5–82.5%), and the Taiwan Island (54.7%) (Hung 2018). The results indicated that cross-boundary transport played a crucial role in the originality and chemical composition of marine PM_2.5_ as compared to local emissions at the Matsu Islands. Even in the summer season, cross-boundary transport from neighboring Fuzhou City and/or upwind sources could contribute approximately two thirds of marine PM_2.5_ at the Matsu Islands. In the seasons of winter and spring, more than four fifths of PM_2.5_ at the Matsu Islands was contributed by cross-boundary transport. The present study strongly suggested that cross-boundary transport from upwind anthropogenic sources contributed major portion of marine PM_2.5_ sampled at the Matsu Islands.

## Conclusions

Both regular and intensive samplings of PM_2.5_ at the Matsu Islands showed that high PM_2.5_ concentrations were mostly observed in winter and spring, particularly during the northeastern monsoon periods, while low PM_2.5_ concentrations commonly occurred in summer. Although the spatial distribution of PM_2.5_ concentration seemed to vary with seasons, the highest PM_2.5_ concentrations were always found at the Nankang Island (NK). It showed very likely that high PM_2.5_ concentration was mainly attributed to cross-boundary transport of PM_2.5_ emitted from anthropogenic sources from either neighboring Fuzhou City or upwind northern regions. Additionally, marine PM_2.5_ sampled at the Matsu Islands was characterized as aged sulfur-rich acidic particles. Secondary inorganic aerosols (SO_4_^2−^, NO_3_^−^, and NH_4_^+^) were the most abundant water-soluble ionic species of PM_2.5_, indicating that the dominant inorganic components of PM_2.5_ were ammonium sulfate and ammonium nitrate. Crustal elements contributed the major portion of metallic content in PM_2.5_ in all seasons, while trace metals appeared apparently during the northeastern monsoon periods, showing that the northeastern monsoons could migrate atmospheric fine particles containing trace metals from the upwind anthropogenic sources to the Matsu Islands. The mass ratios of OC/EC always higher than 2.2 suggested that secondary organic aerosols (SOAs) could be formed during the transport procedure. A further study employing receptor modeling found four major sources of marine PM_2.5_ at the Matsu Islands, namely soil dusts, secondary sulfate, secondary nitrate, and biomass burning. As a whole, this study revealed that cross-boundary transport (CBT) has tremendous impact on the presence of marine PM_2.5_ at the Matsu Islands. More than four fifths of PM_2.5_ at the Matsu Islands was resulted from the cross-boundary transport in the seasons of winter and spring, particularly during the northeastern monsoon periods.
